# Applying T-classifier, binary classifiers, upon high-throughput TCR sequencing output to identify cytomegalovirus exposure history

**DOI:** 10.1038/s41598-023-31013-z

**Published:** 2023-03-28

**Authors:** Kaiyue Zhou, Jiaxin Huo, Caixia Gao, Xu Wang, Pengfei Xu, Jiahuan Hou, Wenying Guo, Tao Sun, Lin Da

**Affiliations:** 1 Department of Mathematics, School of Mathematical Sciences, Inner Mongolia University, Hohhot, China; 2 Hangzhou ImmuQuad Biotechnologies, Hangzhou, China; 3 Institute of Wenzhou, Zhejiang University, Wenzhou, China

**Keywords:** Computational biology and bioinformatics, Immunology, Mathematics and computing

## Abstract

With the continuous development of information technology and the running speed of computers, the development of informatization has led to the generation of increasingly more medical data. Solving unmet needs such as employing the constantly developing artificial intelligence technology to medical data and providing support for the medical industry is a hot research topic. Cytomegalovirus (CMV) is a kind of virus that exists widely in nature with strict species specificity, and the infection rate among Chinese adults is more than 95%. Therefore, the detection of CMV is of great importance since the vast majority of infected patients are in a state of invisible infection after the infection, except for a few patients with clinical symptoms. In this study, we present a new method to detect CMV infection status by analyzing high-throughput sequencing results of T cell receptor beta chains (TCRβ). Based on the high-throughput sequencing data of 640 subjects from cohort 1, Fisher’s exact test was performed to evaluate the relationship between TCRβ sequences and CMV status. Furthermore, the number of subjects with these correlated sequences to different degrees in cohort 1 and cohort 2 were measured to build binary classifier models to identify whether the subject was CMV positive or negative. We select four binary classification algorithms: logistic regression (LR), support vector machine (SVM), random forest (RF), and linear discriminant analysis (LDA) for side-by-side comparison. According to the performance of different algorithms corresponding to different thresholds, four optimal binary classification algorithm models are obtained. The logistic regression algorithm performs best when Fisher's exact test threshold is 10^−5^, and the sensitivity and specificity are 87.5% and 96.88%, respectively. The RF algorithm performs better at the threshold of 10^−5^, with a sensitivity of 87.5% and a specificity of 90.63%. The SVM algorithm also achieves high accuracy at the threshold value of 10^−5^, with a sensitivity of 85.42% and specificity of 96.88%. The LDA algorithm achieves high accuracy with 95.83% sensitivity and 90.63% specificity when the threshold value is 10^−4^. This is probably because the two-dimensional distribution of CMV data samples is linearly separable, and linear division models such as LDA are more effective, while the division effect of nonlinear separable algorithms such as random forest is relatively inaccurate. This new finding may be a potential diagnostic method for CMV and may even be applicable to other viruses, such as the infectious history detection of the new coronavirus.

## Introduction

The rapid growth of information technology and computing speed has led to breakthroughs in hot technologies such as artificial intelligence. Meanwhile, along with the informatization of the medical industry, increasingly more medical data are generated progressively. Questions such as how to apply medical data for disease prevention and detection and how to apply the developing artificial intelligence technology to health care urgently need to be solved, and the problem of how to apply the developing artificial intelligence technology to health care to answer medical questions is a hot topic of current research. Artificial intelligence and its offshoots, such as machine learning (ML), have made significant achievements in the healthcare industry over the past decades, playing a key role in medical diagnosis, and have been practiced through intelligent applications^1^. Some important applications of ML in clinical practice include providing up-to-date information for reducing diagnostic and treatment errors, real-time inference, health risk alerts, and health outcome prediction. Currently, an increasing number of diseases can be identified and initially predicted by machine learning. In 2018, Ocampo et al. built a lung cancer diagnosis model based on neural networks, which achieved an AUC of 0.97 for lung cancer identification^1^, and in 2020, Lyngdoh et al. used multiple algorithms to predict diabetes, with the K-nearest neighbor algorithm achieving an accuracy of 76%^2^. Meanwhile, medical research has begun to shift from traditional disease prevention to cellular and genetic aspects^3^, seeking to discover the root causes of disease eradication. Machine learning algorithms have been used in a wide variety of applications and medical studies. Although there are various types of algorithms, the main algorithms currently in use are still traditional algorithms such as random forests^4^, logistic regression^5^, and support vector machines^6^, which have a sound theory. However, there is no best algorithm for all diseases, and various algorithms need to be used for different diseases.

Cytomegalovirus (CMV) is a herpes virus that is very widely infected in the population, with an infection rate of more than 95% in Chinese adults. After initial infection, CMV remains in a lifelong latent state within the host cells and is in a state of periodic subclinical reactivation under the regulation of the functional immune system. When reactivation or primary infection occurs in patients with severe immune dysfunction, uncontrolled CMV replication leads to clinical manifestations characterized by fever, myelosuppression and tissue-invasive disease^7^. The status of CMV infection can be achieved by analysis of high-throughput sequencing results of the T cell receptor beta chain (TCRβ)^1,8^.

T cells are bone marrow-derived lymphoid stem cells that are distributed to immune organs and tissues throughout the body through lymphatic and blood circulation and have antibody-like molecules called T cell receptors (TCRs) that transmit antigenic signals into T cells. T cell receptors are composed of α and β chains, and TCRs are randomly rearranged through the V, D, and J genes in the genome^9^. The major binding region of antigen, CDR3 (Complementary Determining Region 3), also has random insertions or deletions of nontemplate nucleotides (N-diversity mechanisms) at the junctions of V–D, D–J (beta chain), and V–J gene (alpha chain) fragments, further increasing the diversity of the TCR. Thus, human T cell receptors would have very high diversity, resulting in a theoretical variety as high as 10^12^ ~ 10^18^. In practice, however, it is subject to certain mechanisms (e.g., positional effects, rearrangements are not completely random, and human TCR diversity can reach approximately 10^13^ orders of magnitude). Therefore, next-generation T cell receptor sequencing is an important application for preventive medicine and characterization therapy.

Individuals with healthy immune systems have millions of unique TCRβ chains in circulating T cells. These chains are generated from VDJ rearrangements. The corresponding T cells will then proliferate once they have been exposed to disease antigens. T cell responses to a particular antigen can only be observed in the unexposed compartment of subjects. However, these responses can be detected in the presence of subjects with similar immunological backgrounds.

In this research, the cross-entropy loss and FDR estimate calculated from previously published data^1^ are used to calculate different numbers of feature values corresponding to the P-value thresholds of the logarithmic lattice. The algorithms such as logistic regression and random forest are used to operate by selecting different thresholds and thus different numbers of features, respectively, and the results of multiple algorithms are compared.

## Methods

### Data acquisition and introduction

This article is based on the research data of Emerson et al.^10^. Data are publicly available and downloaded from IMMUNOSEQ ANALYZER (https://clients.adaptivebiotech.com/). Compared with the 666 training samples in the original article, we successfully download 640 samples, including 289 positive samples and 351 negative samples. Different from the 120 test samples in the original article, this article analyzes 112 samples, with 48 positive samples and 64 negative samples. The original data are the specific details of various TCRβ sequences of each sample. This research performs simple data prepossessing and sample data integration and conducts subsequent analysis. A total of 640 training samples in cohort 1 by high-throughput sequencing were pooled. The final TCRβ data had a total of 113,846,476 unique sequences, of which 53,791,329 were from positive subjects and 67,597,252 were from negative subjects.

### Identification of CMV-associated TCRβs

To determine the key sequences that indicate CMV exposure history, we established a confusion matrix for each sequence to calculate their Fisher's exact test p values^11^ as a marker of independence between the sequence and subjects’ CMV status, with the occurrence of CMV-associated TCRβ sequences of the positive and negative groups in cohort 1 as two categorical variables. Therefore, the dependent sequences obtained by Fisher's exact test are only related to training subjects in cohort 1.

### Determination of the best classification threshold

The calculation of AUC is obtained through a combination of multiple classification thresholds, so we evaluate the model classification accuracy according to these thresholds and select the classification threshold with the highest accuracy as the judgment standard of the model^12^. Without this work, the classification model will automatically adopt the default value inside the model, which is not conducive to the effective classification of the model.

### Binary algorithm

Different traditional binary algorithms have been proposed to classify samples from cohort2 into positive and negative groups, corresponding to LR^5^, SVM^6^, RF^13^ and LDA^14^. Before it, biological epistemology is applied to reduce the dimension of multidimensional data, including unique TCRβs (the number of types of TCRβ possessed by each sample) and CMV-associated TCRs (corresponding to different thresholds, the number of sequenced species in which related sequences overlap)^10^. These two indicators of each sample in cohort 1 and their corresponding CMV status were input into four binary classification algorithms to train the model, and CMV exposure history classification of the test sample points and the corresponding indicators such as accuracy, sensitivity, specificity, F1 score, AUC value, and cross-entropy loss function were obtained accordingly.

To obtain the best classification effect of each classification algorithm on this set of binary classification data, we use grid search and traverse each kernel function to classify the data.

For the logistic regression algorithm, parameters such as multi_class as multinomial, solver as newton-cg, class_weight as balanced, and max_iter as 10,000 are selected, and other parameters are default values.

In the parameter adjustment process of the SVM algorithm, we traverse multiple penalty factors C and gamma values of multiple kernel functions (poly, RBF, linear, sigmoid) and obtain the best classification effect when the kernel function is linear, the penalty term C is 1, and the random_state is 10. In addition, to obtain the classification probability and calculate the AUC value, we set the probability equal to True.

In regard to the random forest algorithm, after several parameter adjustments, the combination of n_estimators being 210, max_depth being 5, random_state being 3, class_weight being balanced and OOB_score being True is finally selected, and its test results are obviously better than other combinations. At the same time, to avoid the problem of data overfitting, gini is selected as the criterion in parameter adjustment because the calculation of gini is relatively simple compared with entropy. At the same time, due to the amount of data used, parameters such as min_samples_split, min_samples_leaf and min_weight_fraction_leaf are not reset this time, and the default settings are adopted.

After a series of attempts to find the parameters of linear discriminant analysis, we found that the default combination of parameters makes the model perform well enough.

### Selection of the best cutoff of the P value

Based on the variation in the AUC value, FDR, cross-entropy loss, and F1 score obtained by the trained model with the threshold, we determined the optimal Fisher's exact test threshold for each algorithm. The higher the AUC and F1 score are, the better, and the lower the FDR and cross-entropy loss are, the better. We describe these indexes briefly as follows.

FDR, also known as the positive false discovery rate, means the proportion of samples with errors in judgment among all samples found to be positive, which is the proportion of false (false discovery) in all discoveries calculated^15^. In addition, it means the expected value of the ratio of false rejections (rejecting true hypotheses) to the number of all rejected null hypotheses^16^.

$$FDR = \frac{{FP}}{{FP + TP}}$$

The cross-entropy loss function is also known as the logarithmic loss function, which can be calculated as follows:

$$Cross \,  Entropy \, Loss=-\frac{1}{N}\sum_{i=1}^{N}[{c}_{i}log{q}_{i}(\phi )+(1-{c}_{i})log(1-{q}_{i}(\phi ))]$$

The area under the curve (AUC) is defined as the area under the ROC^17^ curve enclosed by the coordinate axis.

The F1 score can be regarded as a harmonic average of the model's precision and recall, with a maximum value of 1 and a minimum value of 0.

$$F1\,score=\frac{2*TP}{2*TP+FP+FN}$$

In the article, we select four major items as the measurement of four algorithms: accuracy, sensitivity, specificity and Kappa score.

$$Accuracy=\frac{TP+TN}{TP+TN+FP+FN}$$

$$Sensitivity=\frac{TP}{TP+FN}$$

$$Specificity=\frac{TN}{TN+FP}$$

$$Kappa=\frac{Accuracy-{p}_{1}}{1-{p}_{1}}$$

$${p}_{1}=\frac{(TP+FN)*(TP+FP)+(FP+TN)*(FN+TN) }{{(TP+TN+FP+FN)}^{2}}$$

where TP is the number of correctly classified positive samples and TN is the number of correctly classified negative subjects. FP and FN represent the number of falsely classified negative and positive samples, respectively.

## Result

### Identification of CMV-associated TCRs

Fisher’s exact test: Based on the number of samples of each CMV-associated TCRβ that were present in each of the positive and negative samples in training sample cohort 1 and the number of samples without the data of this sequence, the confusion matrix of each sequence was built to calculate the p-value of the Fisher exact test. Therefore, the correlation sequence obtained by Fisher’s exact test is only related to cohort 1 training data and has little direct correlation with test data from cohort 2.

**Figure 1:**
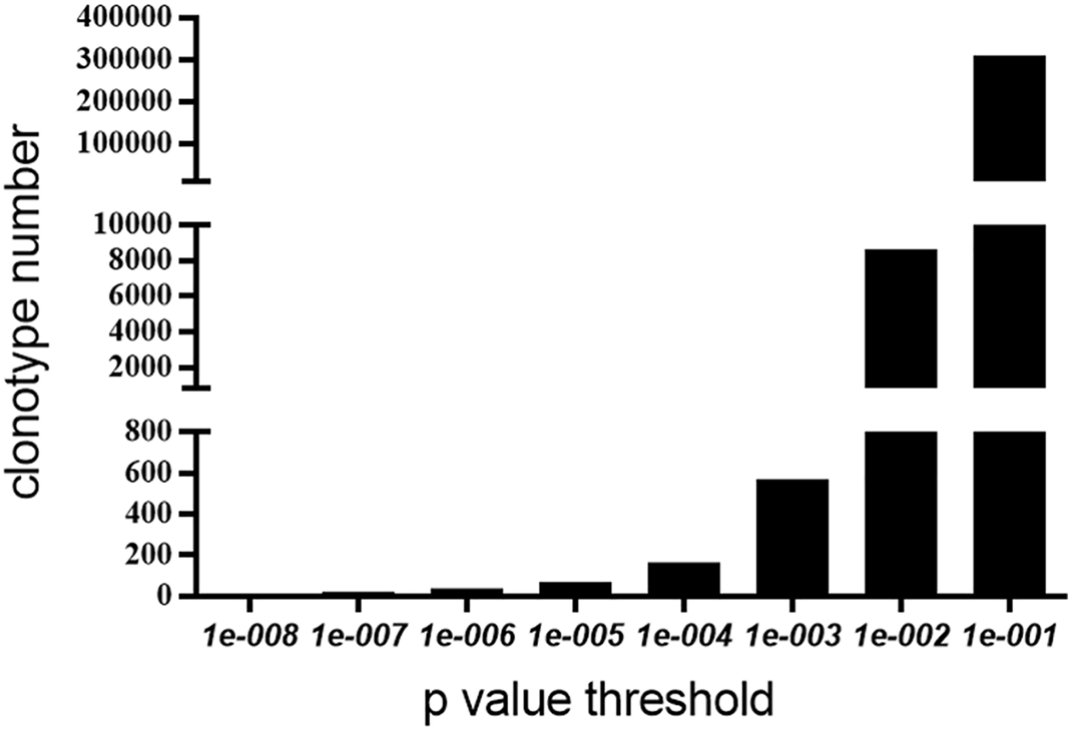
Associated TCRβ sequences. The picture shows the number of CMV-associated TCRβ sequences corresponding to different thresholds of p value (10^−8^ ~ 10^−1^).

The combinations of different thresholds of this P-value and thus different numbers of TCRβ sequences were selected, where the number of CMV-associated TCRβ sequences corresponding to thresholds of 10^−1^, 10^−2^, 10^−3^, 10^−4^, 10^−5^, 10^−6^, 10^−7^, and 10^−8^ were 309,406, 8638, 571, 166, 70, 39, and 11, respectively. Graphs were created based on the threshold value and the number of CMV-associated TCRβ corresponding to this threshold value, as shown in Fig. 1.

### The best cutoff of the p-value for LDA is 10^−4^ and 10^−5^ for LR, SVM and RF

**Figure 2:**
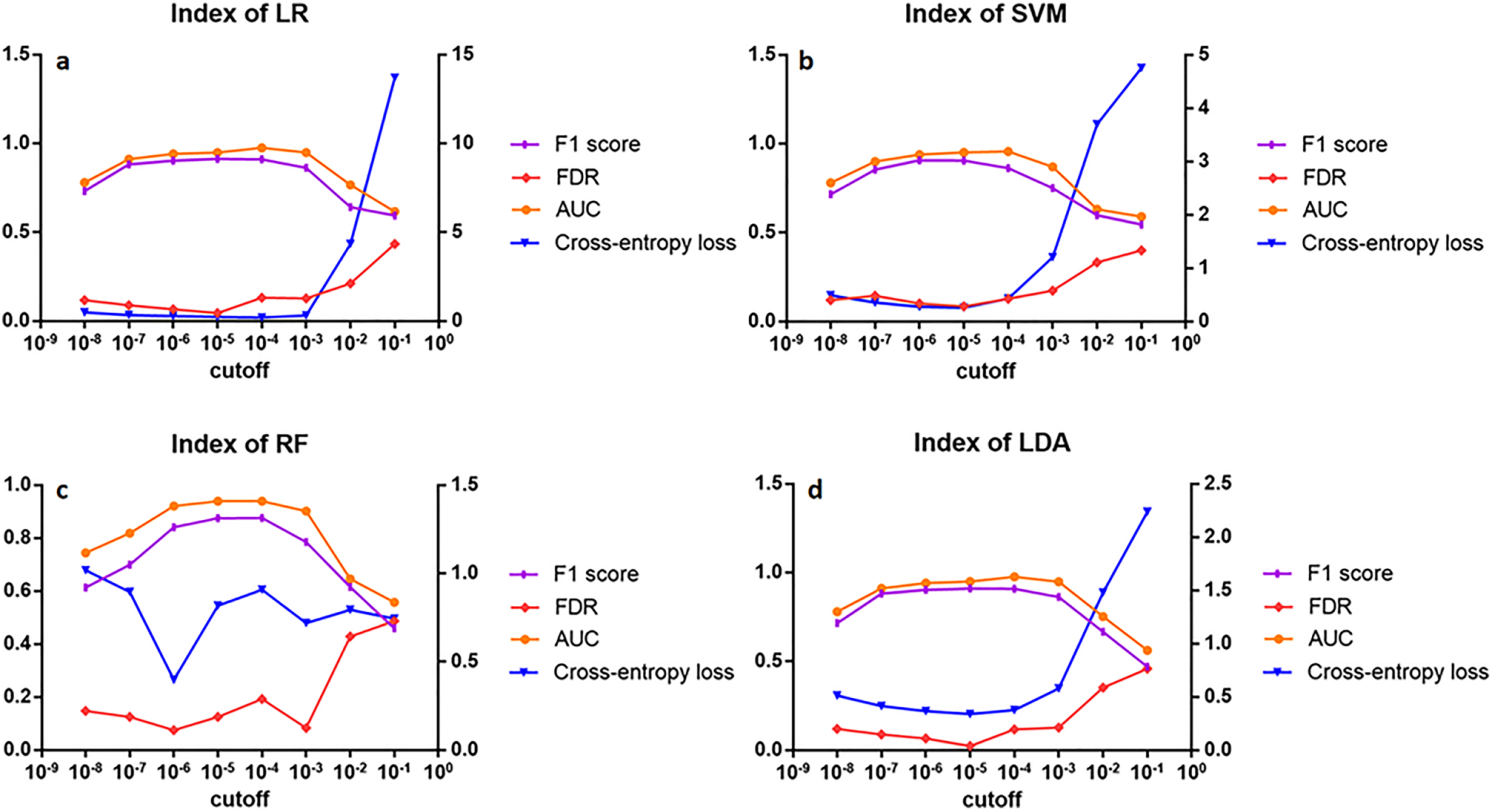
Evaluation metrics of algorithms. The scores of four algorithms of logistic regression (LR, **a**), support vector machine (SVM, **b**), random forest (RF, **c**), and linear discriminant analysis (LDA, **d**) were based on four evaluation metrics. The purple, red, orange, and blue curves represent the F1 score, FDR, AUC, and cross-entropy loss, respectively, with the first three indicators on the left Y-axis and the cross-entropy loss on the right Y-axis.

Figure 2 describes the performance of the four algorithms' classification effectiveness metrics, measuring the data on the four classification algorithms. The x-axis represents different thresholds of the P value, and the y-axis donates the algorithms with different thresholds corresponding to the four evaluation metrics. As shown in Fig. 2a, the F1 score of the LR logistic regression algorithm presents a trend of first rising and then falling with the increase of the threshold, and it is at a higher level at the thresholds of 10^−4^, 10^−5^, and 10^−6^. When the threshold increases, the AUC value first increases at the beginning and then decreases with increasing threshold, and it is higher at thresholds of 10^−3^, 10^−4^, and 10^−5^ and the highest at 10^−4^. The FDR false discovery rate first decreases and then increases with increasing threshold and is the lowest at 10^−5^. The cross-entropy loss function of LR decreases slowly at first and then increases rapidly with the increase of the threshold, the lowest at 10^−4^, and the second at 10^−5^. Figure 2b shows that the F1 score of the SVM algorithm first increases and then decreases with the increase of the threshold, and it is at a higher level at the thresholds of 10^−4^, 10^−5^, and 10^−6^. The AUC value first increases and then decreases with increasing threshold, and it is higher at thresholds of 10^−4^ and 10^−5^. The FDR error detection rate first increases, then decreases and then increases with increasing threshold and reaches the highest level of 0.0851 at 10^−5^. The cross-entropy loss function of SVM first decreases and then increases with the increase of the threshold, and it is the lowest at 10^−4^ and as low as 0.2609. Figure 2c depicts the trend of the RF random forest algorithm with different thresholds than the previous two algorithms. The F1 score and AUC still increase in the beginning and then decrease with the increase of the threshold and are at higher levels at the intermediate thresholds of 10^−4^, 10^−5^, and 10^−6^. Both the FDR false discovery rate and the cross-entropy loss function fluctuate as the threshold increases and are lower at 10^−3^, 10^−4^, 10^−5^, and 10^−6^. Figure 2d depicts that the F1 score and AUC of the LDA linear discriminant analysis first increase and then decrease with the increase of the threshold and are at higher levels at the intermediate thresholds of 10^−4^, 10^−5^, and 10^−6^, and both achieved the highest level at 10^−4^. Similarly, the FDR and loss function first decrease and then increase with increasing threshold, and both take the lowest value at 10^−5^.

### Decision boundaries for 4 algorithms

**Figure 3:**
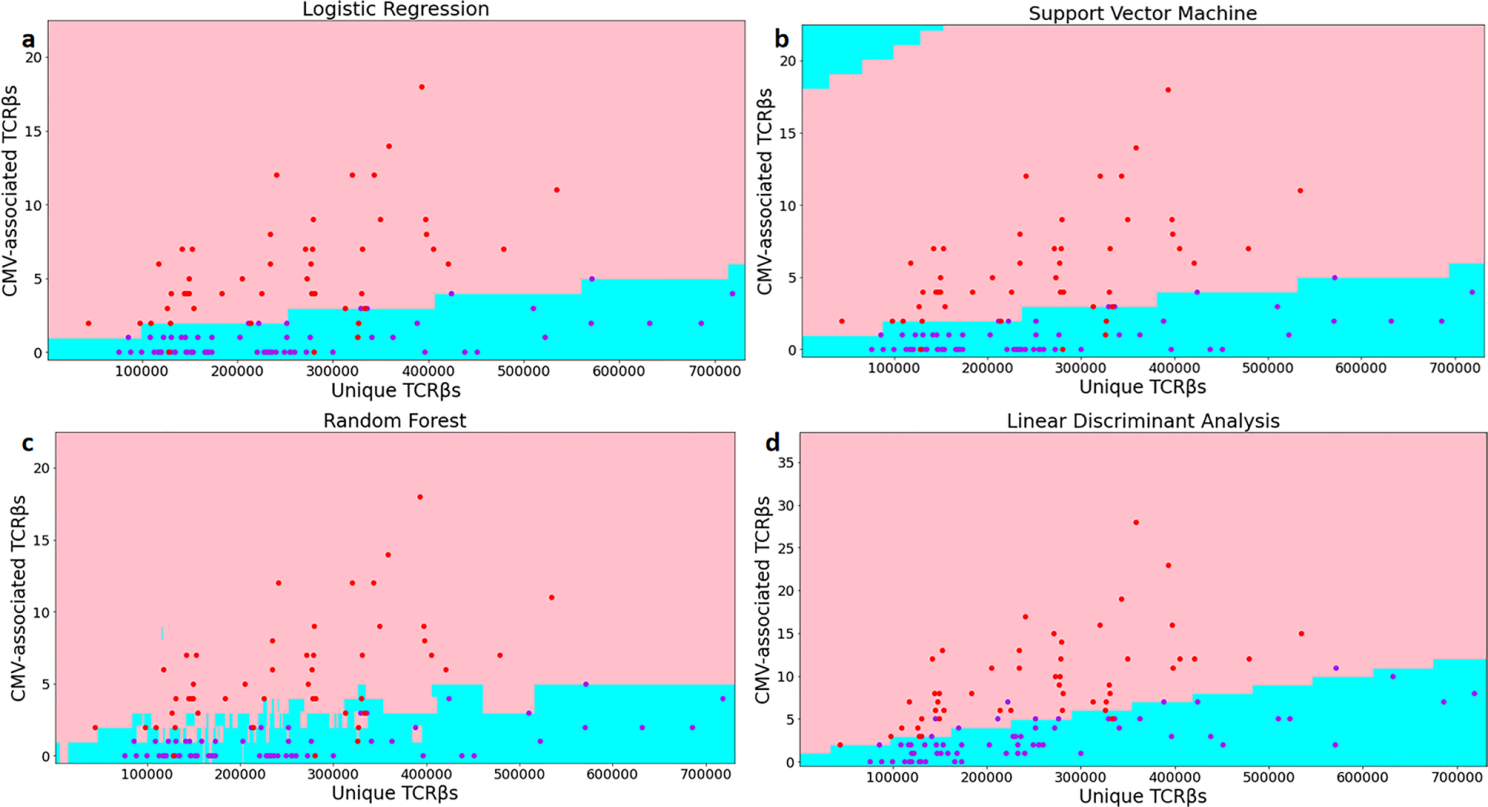
Scatter plots and area classification lines of testing samples. The figure depicts a scatter plot of positive and negative decision boundaries obtained by the four classification algorithms trained on cohort1 training samples and cohort2 test samples, where the x-axis represents the total number of TCRβ sequence species per sample and the y-axis donates the number of repeat species with associated TCR sequences. The blue dots represent negative samples in cohort 2, and the red dots represent positive samples in cohort 2. The pink and sky blue regions represent the positive and negative regions obtained by training each algorithm's cohort1 training data, respectively. Figure (**a**–**d**) shows the classification graph algorithms and the distribution of test sample points of the LR, SVM, RF and LDA, respectively.

As shown in Fig. 3, if a point falls into the pink area, it means that the algorithm predicts positive; otherwise, in the sky-blue area, it means negative. Figure 3a is the classification diagram of the logistic regression algorithm. The segmentation line is presented as an upward sloping straight line. The segmentation effect is good, and the wrongly classified test sample points are not easy to see with the naked eye. Figure 3b shows the classification graph of the SVM algorithm and the distribution of test sample points. Due to the use of a polynomial kernel function with better performance, the area divided by the SVM algorithm contains a certain degree of the circular structure, and there are still some areas in the upper left corner that are classified as negative. Figure 3c is the classification diagram of the random forest algorithm. The overall division of the image seems to be too fitting, and it is easy to cause the model to perform well on the training samples but poorly on the test samples. The classification diagram of the linear discriminant analysis algorithm shown in Fig. 3d is closer to a straight line than the LR algorithm. The division is more detailed and smoother, with a more robust classification effect.

We also depicted the distribution of training samples from cohort 1 on the area with the decision boundaries, as shown in Supplementary Fig. 1.

### The best performance of each algorithm

We support that the best cutoffs of Fisher’s exact test’s p value learning from Fig. 2 are appropriate in Supplementary Fig. 2, with 10^−4^ for LDA, 10^−5^ for SVM and RF, and 10^−4^ or 10^−5^ for LR (Supplementary Fig. 2).

**Figure 4:**
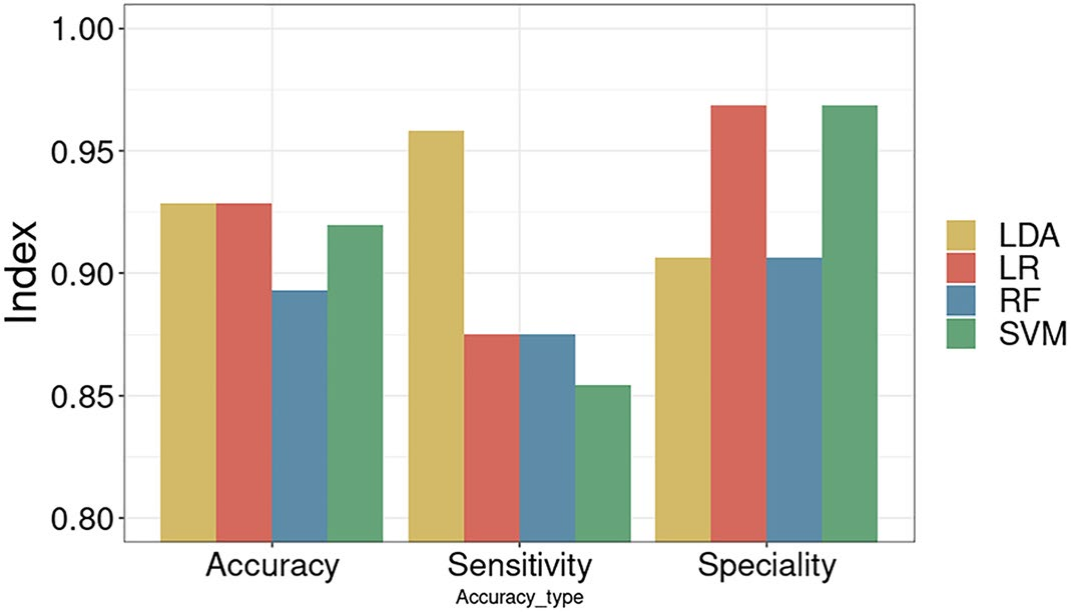
LDA and LR perform better on CMV data. Figure depicts the optimal performance of each algorithm corresponding to the optimal threshold value to obtain the accuracy, sensitivity and specificity of each algorithm.

With the best cutoffs above, Fig. 4 shows the accuracy, sensitivity and specificity of each algorithm according to the best performance corresponding to the best threshold for each algorithm. The three coordinates of the X-axis are accuracy, sensitivity and specificity from left to right, and the algorithms LDA, LR, RF, and SVM are labeled with different colors. There is no significant difference in the accuracy of the four algorithms under the optimal threshold, and the accuracy rates are all nearly above 90%. The accuracy rates of LR and LDA are the highest, reaching 92.86%, followed by SVM with 91.96% and RF with the lowest rate of 89.29%. In terms of sensitivity, LDA performed well, reaching more than 95.83%, and the other three algorithms were in the range of 85% to 88%, indicating that LDA was more inclined to classify samples as positive based on the higher classification accuracy. In terms of specificity, the LR and SVM algorithms performed better, both 96.88%, indicating that these two algorithms were more inclined to classify samples as negative.

From the aspect of measuring the effect of classification from consistency, the Kappa coefficients of LR, SVM, RF and LDA are 0.8346, 0.8364, 0.8518 and 0.8364, respectively. This means that the predicted results of the four models are consistent with the actual classification results.

## Discussion

### Algorithm discussion

Sensitivity is the degree of sensitivity to the detection of positive samples, specificity is the degree of sensitivity to negative samples, and accuracy is a broad measure of accuracy. Sensitivity cannot be overemphasized, nor specificity overemphasized. Overemphasizing the importance of sensitivity will easily make the classifier too sensitive and increase the false positive rate of actual negatives. Conversely, overemphasizing specificity will easily make the classifier too conservative, and a large number of positives may be missed. Accuracy, sensitivity, and specificity are all affected by the judgment threshold, so we selected the best judgment thresholds through AUC to classify the model and calculate the corresponding accuracy rates^18^.

F1 score, AUC, FDR, and cross-entropy loss in Fig. 2. The accuracy, sensitivity, and specificity in Supplementary Fig. 2 are useful for visualizing the performance of classifiers and selecting optional classifiers.

From Fig. 2, the models with higher F1 score and AUC or with lower FDR and cross entropy loss are better classification models. In addition, higher accuracy, sensitivity or specificity represents a better classification effect as well^18^.

It can be seen from the foregoing that the accuracy rates of the LDA and LR algorithms are tied for the highest, but the difference in sensitivity and specificity of the LR algorithm is larger than that of LDA. When the accuracy of LDA is the highest, both sensitivity and specificity can be considered so that both are at a high level at the same time. Therefore, for shuffling data, the best-performing algorithm among the four algorithms is the LDA algorithm. The main reason is that the division of the data is more linearly separable, so the LDA algorithm is a more suitable choice. If it is linearly inseparable, the LDA algorithm is unlikely to be the best-performing algorithm.

### Research evaluation

As mentioned above, our article creatively applied these four binary classification algorithms to a two-dimensional TCRβ array after high-throughput sequencing to diagnose the infection history of cytomegalovirus. It turns out that this methodology is very effective in achieving this goal. The overall logic can be self-consistent. In addition, we tried different algorithms and different parameter adjustment methods (such as calculating the accuracy rate with the best judgment threshold determined by AUC). Consequently, the accuracy rate of the results is greatly improved, and the average accuracy rate reaches more than 90%.

However, there are still many ensemble learning algorithms or neural network algorithms that can obtain better results. Whether multidimens scientific research exploration can be attempted in future articles, such as whether other binary classification algorithms such as the ensional classification algorithm can be tried to obtain the most critical TCR sequence for the expression of this disease, and whether the methodology in this paper is also applicable to other diseases.

## Conclusion

In our article, four binary classification algorithms were proven to achieve excellent performance in diagnosing CMV exposure history with subjects’ unique TCRβ and CMV-associated TCRβ. From the perspective of the AUC evaluation dimension, LDA performs better in the two-dimensional array of CMV virus than the other three algorithms.

In summary, our current study revealed an important signal that linear division models such as LDA are more effective, while the division effect of nonlinear separable algorithms such as random forest is relatively inaccurate, probably because the two-dimensional distribution of CMV data samples is linearly separable^19^. Importantly, the T-classifier may be a potential diagnostic method for CMV and even other viruses.

## Supplementary Information

Supplementary Figures.

## Data Availability

The data that support the findings of this study are available from the Adaptive Biotechnologies immuneACCESS, https://doi.org/10.21417/B7001Z.
