# Supplementary material for: Applying T-classifier, binary classifiers, upon high-throughput TCR sequencing output to identify cytomegalovirus exposure history
Source: Sci Rep. 2023 Mar 28;13:5024. doi: 10.1038/s41598-023-31013-z (PMC10043529; doi:10.1038/s41598-023-31013-z)
Supplement: Supplementary file 1 — Supplementary Figures. [file 41598_2023_31013_MOESM1_ESM.docx]

**Applying T-classifier, binary classifiers, upon high-throughput TCR sequencing output to identify cytomegalovirus exposure history**

Kaiyue Zhou^1*^, Jiaxin Huo^1*^, Caixia Gao^1^, Xu Wang^1^, Pengfei Xu^2^, Jiahuan Hou^2^, Wenying Guo^2^, Tao Sun^2-3*^, Dalin^1*^

**Authors’ affiliation:**

1 Department of Mathematics, School of Mathematical Sciences, Inner Mongolia University, Hohhot, China

2 Hangzhou ImmuQuad Biotechnologies, Hangzhou, China

3 Institute of Wenzhou, Zhejiang University, Wenzhou, China

* Corresponding Authors:

Tao Sun, PhD

Hangzhou ImmuQuad Biotech, LLC., Hangzhou, China

Institute of Wenzhou, Zhejiang University, Wenzhou, China

Telephone: 86-15982358665

1. mail: [taosun@immuquad.com](mailto:taosun@immuquad.com)

Dalin, PhD

Department of Mathematics, School of Mathematical Sciences, Inner Mongolia University, Hohhot, China

Telephone: 86-15647156090

E-mail: 111977331@imu.edu.cn

**Keywords:** Cytomegalovirus，T Cell Receptor, Support Vector Machine，Logistic Regression , Random Forest，Linear Discriminant Analysis

**Additional File:**

File contains supplementary figures from Supplementary Figure 1 to Supplementary Figure 2.

**Acknowledgement and funding**

This work was supported by the Fundamental Research Funds for the Inner Mongolia University(21100-5187051).

**Abbreviations**

**LR:** Logistic Regression

**SVM:** Support Vector Machine

**LDA:** Linear Discriminant Analysis

**RF:** Random Forest

**TCR:** T-cell receptors

**CMV:** Cytomegalovirus

**CDR3:** Complementary Determining Region 3

**D-J:** beta chain

**V-J gene:** alpha chain

**TCRβs:** T-cell receptors beta chain

**AUC:** Area Under Curve

**Availability of data and materials**

The data that support the findings of this study are available from the Adaptive Biotechnologies immuneACCESS, https://doi.org/10.21417/B7001Z

**Declarations**

**Competing interests**

The authors declare no competing financial interests.

**Consent for publication**

All authors have approved the manuscript for submission

**Authors’ contributions**

KZ, CG, XW, PX, JH and WG processed the primary data. KZ and JH built the ML algorithms for the study and drafted the manuscript. LD and TS designed the study and revised the manuscript. All authors checked and approved the final manuscript.

**Authors’ information**

Kaiyue Zhou, Jiaxin Huo, Caixia Gao, Xu Wang & Dalin

Department of Mathematics, School of Mathematical Sciences, Inner Mongolia University, Hohhot, China

Pengfei Xu, Jiahuan Hou, Wenying Guo & Tao Sun

Hangzhou ImmuQuad Biotechnologies, Hangzhou, China

Tao Sun

Institute of Wenzhou, Zhejiang University, Wenzhou, China

**Figure legends**

**Supplementary Figure 1. Scatter plots and area classification lines of training samples.** This figure is the scatter diagram of positive and negative decision boundaries and Cohort1 training subjects obtained by each algorithm according to Cohort1 training subjects.The blue dots represent the negative subjects in Cohort1, and the red dots represent the positive subjects in Cohort1.The pink area and sky blue area respectively represent the positive area and negative area obtained by each algorithm training Cohort1 training data. If a point falls in the pink area, it means that the algorithm predicts it to be positive; otherwise, it means that the sky blue area represents negative.

**Supplementary Figure 2. The evaluation scores of four algorithms.** Supplementary Figure 2 shows the evaluation scores of four algorithms of Logistic Regression (LR, Supplementary Figure 2a), Support Vector Machine (SVM, Supplementary Figure 2b), Random Forest (RF, Supplementary Figure 2c), and Linear Discriminant analysis (LDA, Supplementary Figure 2d). The red, blue and green curves are on behalf of Accuracy, Sensitivity, Speciality respectively.


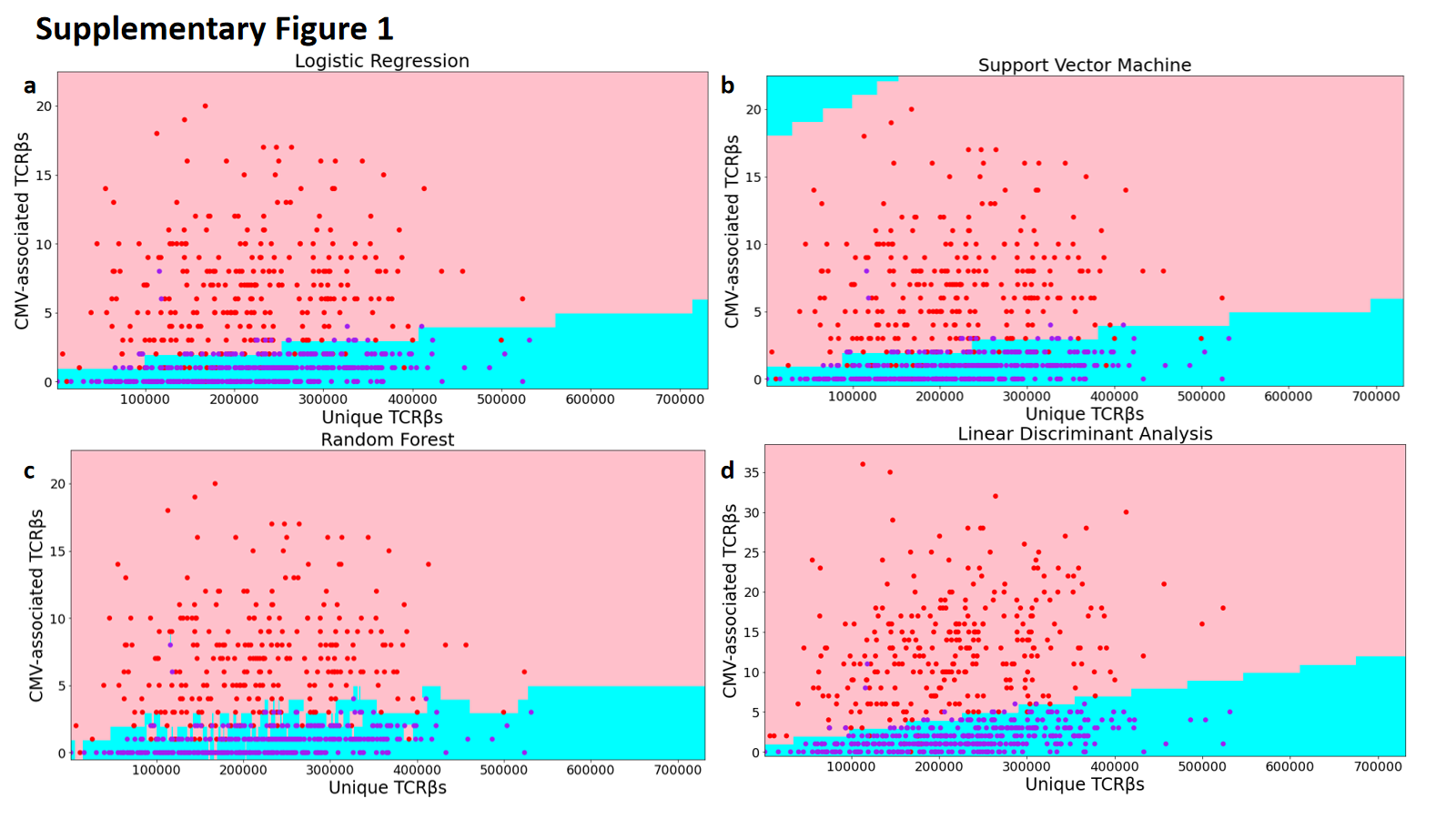


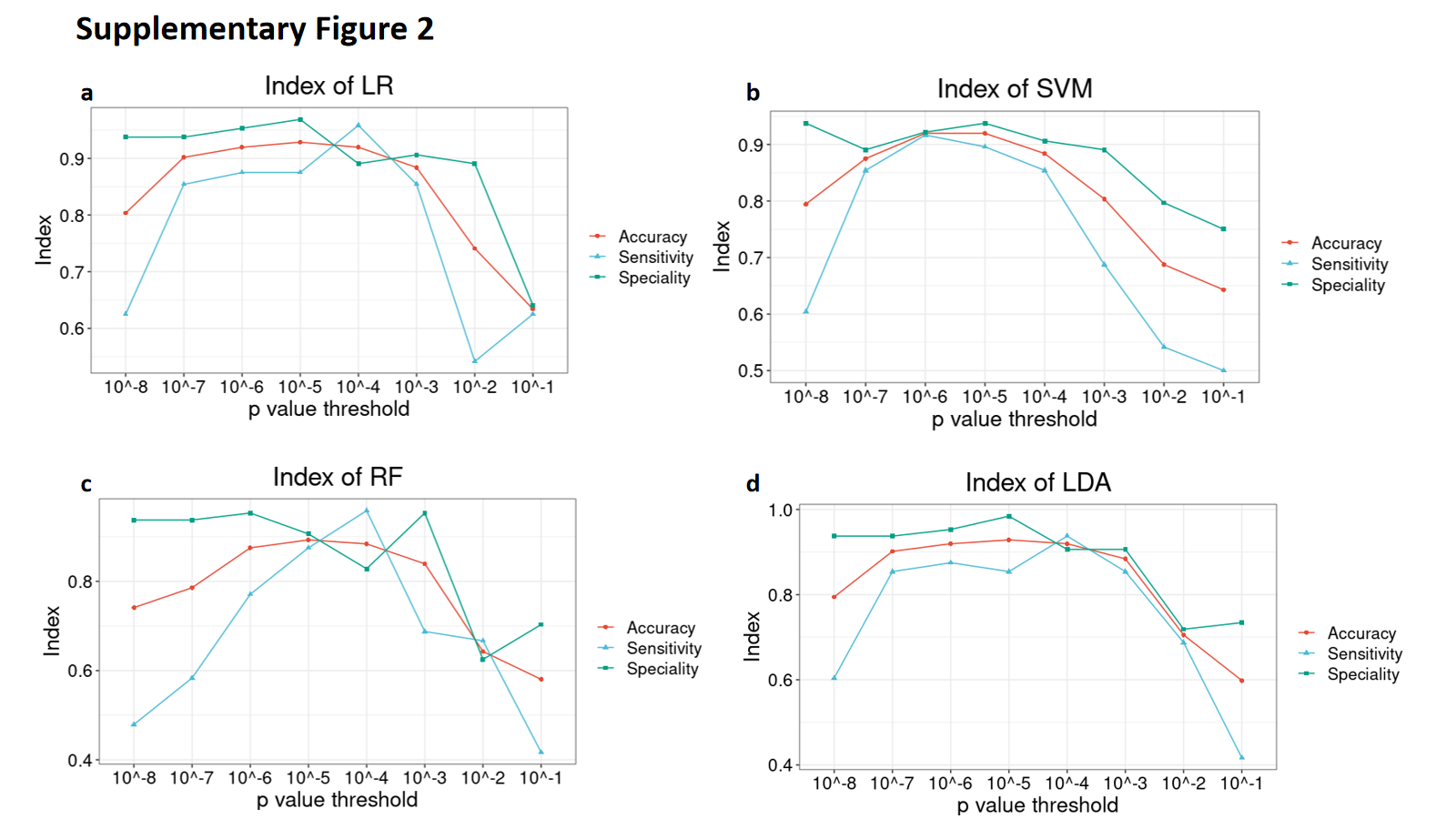


**Tables**

N/A

**Additional Files**

N/A
